# Gendered moral economies of sympathy after suicide in Arabic-language Facebook comments

**DOI:** 10.3389/fsoc.2026.1958676

**Published:** 2026-10-28

**Authors:** Dina Naser Tahat, Khawlah M. AL-Tkhayneh, Emad Mohammad Alghazo, Ismaael AlMazaedh, Khalaf Mohammad Tahat

**Affiliations:** 1 Department of Applied Sociology, College of Education, Humanities and Social Sciences, Al Ain University, Al Ain, United Arab Emirates; 2 College of Education, Humanities and Social Sciences, Al Ain University, Al Ain, United Arab Emirates; 3 Department of Arabic Language and Literature, United Arab Emirates University, Al Ain, United Arab Emirates; 4 Department of Arabic Language, The University of Jordan, Amman, Jordan; 5 Department of Media and Creative Industries, United Arab Emirates University, Al Ain, United Arab Emirates; 6 Department of Journalism and Digital Media, Faculty of Mass Communication, Yarmouk University, Irbid, Jordan

**Keywords:** Arab digital publics, digital death discourse, gender, moral economy, social media, sociology of emotions, suicide stigma, sympathy

## Abstract

**Introduction:**

This study examines sympathy as a socially organized emotion in Arabic-language digital discourse about suicide. Drawing on the sociology of emotions, suicide stigma, and digital death studies, it investigates how public Facebook comment cultures distribute compassion, blame, and moral worth after suicide.

**Methods:**

A quantitative content analysis was conducted on 2,132 publicly visible comments responding to three highly discussed suicide cases. Comments were coded for inferred public gender presentation, emotional orientation, reported case context, and dominant interpretive frame. Pearson chi-square tests were calculated from raw counts; Cramer’s *V*, 95% Wilson confidence intervals, and sensitivity analyses are reported.

**Results:**

Comments from accounts inferred as female were more often sympathetic than comments from accounts inferred as male (60.0% vs. 40.5%; *V* = 0.15), and commenter composition differed across the three cases (*V* = 0.29). Religious framing was the most common interpretive repertoire (48.6%) and could accompany both mercy and condemnation.

**Discussion:**

The study develops the concept of a moral economy of sympathy to describe the social rules through which digital publics allocate compassion after suicide. Because each case combines a particular person, national setting, reported circumstance, and media framing, the findings are case-specific and should not be generalized to Arab publics.

## Introduction

Suicide is a major global public health and social concern. The World Health Organization (2025) estimates that more than 720,000 people die by suicide each year, and suicide is shaped by multiple social, psychological, economic, cultural, and institutional factors. Yet suicide is also a public death. Once reported by news media and circulated through social media, it becomes an occasion for collective interpretation: audiences mourn, condemn, speculate, assign blame, demand justice, or deny sympathy.

This article approaches public responses to suicide news as digital death discourse: mediated textual interaction through which users interpret death, assign meaning to the dead, and negotiate obligations of mourning, condemnation, prevention, and justice. Research on mediated death and online mourning shows how digital environments reshape encounters with death and public mourning (Klass et al., 1996; Walter et al., 2012; Brubaker et al., 2013; Sumiala, 2022). Klastrup (2015, 2018) examines mourning by dispersed publics and the importance of temporal context, Harju (2015) addresses socially shared mourning and collective memory, and Stokes (2015) considers the moral status of digital remains. More recent work by Harju and Pentikäinen (2025) further shows how public grief is shaped by platform conditions. These studies help situate the present focus on how dignity, sympathy, and condemnation are negotiated in public news-comment settings.

Facebook comment sections are also shaped by platform processes rather than functioning as transparent records of public opinion. Bucher (2012) shows how visibility is algorithmically organized; (Gillespie 2018) emphasizes the role of moderation in determining what remains available; and (van Dijck et al., 2018) situate such selection within the wider organization of platform society. The present study therefore treats the observed comments as platform-conditioned public expressions and does not assume that they represent every response generated by the posts.

A sociology-of-emotions perspective shifts the analytic question from whether sympathy is present to how sympathy becomes socially organized. Emotions are not only private psychological states; they are relational, culturally patterned, and linked to social expectations about what people should feel and toward whom. Hochschild's concept of feeling rules identifies socially shared standards of what is emotionally appropriate in a situation and to a particular object (Hochschild, 1979). More broadly, the sociology of emotions emphasizes the links among feeling, culture, social position, interaction, and collective life (Bericat, 2016). From this perspective, sympathy after suicide can be studied as a socially regulated form of recognition rather than simply as an individual disposition.

Arab digital spaces provide an important setting for this analysis. Public discussion of suicide in many Arabic-language environments takes place within overlapping religious, familial, gendered, and institutional moral orders. Suicide is religiously prohibited in dominant Islamic discourse, mental health problems remain stigmatized in many settings, and public debates around gender, sexuality, sexual violence, work pressure, and state responsibility are socially charged. Research on digital religion further shows that religious communication online is shaped by platform infrastructures, actor strategies, discourse, and audience participation, and can reinforce both cohesion and exclusionary boundary-making (AlMazaedh et al., 2026). Social media platforms make these judgments immediately visible and allow ordinary users to participate in the public definition of a death.

Prior research on suicide-news comments has found mixtures of sympathy, anger, ridicule, speculation, moral judgment, and institutional critique (Sisask et al., 2005; Rosen et al., 2020; Al-Zaman and Rashid, 2022). Research in Egypt has also examined the role of social media in forming public stereotypes of suicide (Mahmoud, 2022). Media and social-media research indicates that the identity of the person who died and the perceived circumstances of death can shape public response. In the case of Sarah Hegazi, for example, comparative media research identified strong stigma frames and uneven sympathy across national contexts (Al Ajjan and Kozman, 2023). Suicide-related stigma can appear through condemnation, ridicule, denial of mercy, exclusion of the deceased from moral community, or blame directed toward families and survivors (Link and Phelan, 2001).

Gender is also relevant to public responses to suicidality. Studies in several contexts have found gender differences in emotional involvement, willingness to help, and attitudes toward suicide (Dahlen and Canetto, 2002; Eskin et al., 2016; Bezerra et al., 2022; Chen et al., 2025). The present study does not treat such differences as fixed psychological traits. Instead, inferred gender presentation is examined as a social position that may be associated with different forms of proximity to vulnerability, injustice, care, work, provision, and respectability. The analytic emphasis is therefore on patterned emotional allocation rather than presumed essential differences between men and women.

The concept of moral economy sharpens this approach. Fassin (2009) uses moral economy to analyze the production, circulation, and distribution of values, norms, obligations, emotions, and moral sentiments in social space. Applied to suicide comments, a moral economy of sympathy refers to the patterned production, circulation, distribution, and withholding of compassion in public responses to suicide deaths. Sympathy is allocated through socially available scripts of innocence, shame, injustice, respectability, religion, gender, and responsibility. In Hochschild's terms, these scripts can be understood as feeling rules concerning who may be mourned, how much compassion is appropriate, and when condemnation displaces sympathy.

Digital death discourse is the setting in which this emotional allocation becomes publicly observable. Religious language may become either mercy or exclusion; grief may become institutional accusation; and news comments may turn death into an arena of symbolic boundary-making. Durkheim's theory of suicide provides one lens for comments that attribute death to weak support systems, work pressure, institutional neglect, or social exclusion by locating suicide within problems of integration and regulation. Bourdieu's concepts of symbolic power and doxa provide another: comments that deny sympathy on the basis of sexuality, gendered respectability, religion, or perceived weakness reproduce taken-for-granted boundaries of legitimate suffering and moral worth (Durkheim, 1951; Bourdieu, 1991).

The present study analyzes 2,132 Facebook comments responding to three suicide deaths that received extensive attention in Arabic-language news media: an Egyptian LGBTQ + activist, a Moroccan minor whose death followed reported sexual violence and perceived judicial injustice, and an Egyptian employee whose death was publicly discussed in relation to workplace and economic pressure. The cases are not treated as clinically verified causes of suicide. They are purposively selected public cases around which commenters organized sympathy, stigma, blame, and responsibility.

The study asks four questions: (RQ1) How do Arabic-language Facebook commenters express sympathy, stigma, and moral judgment in response to suicide news? (RQ2) How is inferred commenter gender presentation associated with sympathetic, unsympathetic, and unclear responses? (RQ3) What interpretive frames structure public responses to these suicide cases? (RQ4) How do online comments distribute or withhold moral worth after suicide? Two hypotheses were tested: H1, emotional orientation differs by inferred commenter gender presentation; and H2, the inferred gender composition of commenters differs across the three cases.

## Materials and methods

### Design

The study used quantitative content analysis to examine public Facebook comments on Arabic-language suicide news. The individual top-level comment was the unit of analysis. Content analysis was selected because the research questions concern patterned emotional orientations, moral classifications, and interpretive frames across a large corpus of user-generated comments, and because public news comments can provide systematic data for social inquiry when their contextual limits are made explicit (Henrich and Holmes, 2013).

### Case selection and data sources

Three suicide deaths were purposively selected because they generated substantial public attention and were publicly associated with distinct narratives: stigma and marginalization, sexual violence and perceived injustice, and workplace/economic pressure. The selection was not designed to represent Arab publics. Each narrative corresponds to one person and one case, so context, gender of the deceased, nationality, public identity, and media framing vary together. Cross-case differences therefore describe these particular case configurations and cannot identify the independent effect of any one attribute. Table 1 summarizes the case features and these sources of confounding.

**Table 1:** Comparison of case features and inseparable sources of confounding.

Coded case	Documented identity/Setting	Reported context/Media framing	*n*	Attributes that vary with the case
Case 1: stigma/marginalization	Egyptian woman; LGBTQ+ activist; death in Canada	Stigma, marginalization, and public identity	1,081	Gender, sexuality, activist status, migration/exile, national setting, and news presentation
Case 2: discrimination/injustice	Identified in the submitted study as a Moroccan minor girl	Reported sexual violence, stigmatization, and perceived judicial injustice	761	Minor status, gender, violence, legal narrative, national setting, and news presentation
Case 3: work/economic pressure	Egyptian man; employee; workplace setting	Reported work pressure and economic strain	290	Gender, employment status, economic role, national setting, and news presentation

Each reported context is represented by a single case. The comparison makes the bundled attributes explicit; it does not isolate causal effects and does not support population generalization.

The first case concerned Egyptian LGBTQ + activist Sarah Hegazi, who died in Canada after reported detention, torture, exile, and public stigma following participation in an LGBTQ + rights event. The second concerned a Moroccan minor who died after reported kidnapping, repeated sexual assault, public stigmatization, and perceived failure of judicial accountability. The third concerned an Egyptian employee who died at his workplace amid public reports of work pressure, economic stress, and dismissal. The analysis concerns comments responding to news coverage of these deaths and does not make clinical determinations about individual causality or mental health status.

### Data collection and cleaning

The dataset consisted of publicly visible Facebook comments posted under news items published by BBC Arabic and Al Jazeera Arabic. Comments were extracted by the research team from public news pages in 2023. Replies, duplicate comments, tagging-only comments, spam, commercial links, comments consisting only of emojis, and unrelated material were excluded. The final dataset included 2,132 top-level comments: 1,081 on the activist case, 761 on the Moroccan minor case, and 290 on the employee case.

### Platform visibility, temporal scope, and media framing

The comments analyzed here are the comments that were visible and retained at collection. The archived screenshots show different Facebook display modes, including Most relevant in the activist capture and All comments in other captures. These settings, together with moderation and deletion, may shape what is visible; they do not reveal the full ranking history or content no longer available. The study therefore treats the corpus as platform-conditioned rather than as a transparent record of all responses.

The archived posts preserve publication dates, but the analytic data do not retain exact comment timestamps linked to coded records. The elapsed time between each death and each analyzed comment cannot therefore be reconstructed, so the study cannot examine how emotional responses evolved over time. Media framing is summarized at the case level in Table 1. Because each framing configuration corresponds to one case and there is no within-case variation in news framing, its independent effect on comments cannot be estimated.

### Coding framework

#### Inferred public gender presentation

Commenter gender presentation was coded as male, female, or undetermined using the Arabic first name or public username displayed with the comment. The documented coding examples included Fatima, Souad, and Ahlam as feminine cues and Muhammad, Khalid, and Ahmad as masculine cues. Usernames beginning with Umm were treated as feminine-presenting and those beginning with Abu as masculine-presenting. Ambiguous or regionally variable names, nonpersonal or organizational accounts, decorative phrases, pseudonyms, shared-family handles, and names that did not support a confident classification were coded as undetermined rather than forced into a binary category. Latin-script names were classified only when the naming cue remained clear. Profile photographs, biographies, and private information were not used. The measure therefore captures inferred public gender presentation rather than verified gender identity, and regional variation or pseudonymous naming may still produce misclassification.

#### Emotional orientation

Emotional orientation toward the person who died was coded as sympathetic, unsympathetic, or unclear. Sympathetic comments expressed mercy, grief, compassion, recognition of injustice, or concern for the deceased. Unsympathetic comments condemned the deceased, denied mercy, blamed the person who died, mocked the death, or treated the death as deserved. Unclear comments were ambiguous, unrelated, or insufficiently interpretable.

#### Reported case context

The three cases were categorized according to their dominant public framing: social stigma and marginalization, discrimination and injustice, and work/economic pressure. These categories describe how the cases were publicly organized in news and comment discourse; they are not clinical explanations of the deaths. Because each context corresponds to one case, context and case cannot be statistically disentangled in this design.

#### Dominant interpretive frame

The dominant interpretive frame of each comment was coded as religious, state/institutional, social, victim-blaming, or unclear. Religious comments invoked Quranic verses, hadith, supplication, sin, divine judgment, mercy, patience, or religious prohibition. State/institutional comments placed responsibility on courts, police, employers, government, media, or mental health systems. Social comments emphasized family, customs, stigma, gender relations, social cohesion, or community values. Victim-blaming comments placed primary responsibility on the person who died. Unclear comments did not contain enough relevant information to classify.

Emotional orientation and interpretive frame were coded as separate analytical dimensions. A religious comment could therefore be coded as sympathetic when it expressed prayer, mercy, or forgiveness, or as unsympathetic when it invoked condemnation, prohibition, or denial of mercy. The religious category captures the dominant vocabulary through which a comment interprets the death rather than a single emotional valence. This distinction permits analysis of religious discourse as an ambivalent cultural repertoire while preserving the separate measure of emotional orientation.

### Arabic-language coding procedure

Comments were coded in their original Arabic. Coders read the full comment rather than isolated keywords and considered colloquial spelling, dialectal vocabulary, negation, quoted speech, religious formulae, code-switching, emojis used with text, and pragmatic tone. Sarcasm and irony were interpreted from the stance of the whole statement. When dialect, humor, ellipsis, or mixed signals made emotional orientation or dominant frame uncertain, the relevant variable was coded as unclear rather than forcing a judgment. The English examples below were prepared only for reporting and were not used in the original category assignments.

### Validity and intercoder reliability

The coding scheme was developed after an initial reading of the comments and was reviewed by faculty members in sociology, psychology, and education. Category boundaries were revised to reduce overlap. Intercoder agreement was assessed in the original study using Holsti's formula, with coefficients of 0.90 for the main variables collectively, including reported case context, inferred gender presentation, and emotional orientation, and 0.83 for dominant interpretive frame. Disagreements were discussed and resolved before the final analysis. Holsti's coefficient reports observed agreement rather than chance-corrected agreement. Because Cohen's kappa or Krippendorff's alpha cannot be derived validly from the reported aggregate Holsti coefficients alone, no retrospective chance-corrected coefficient is estimated here.

### Data analysis

Frequencies and percentages were calculated for all variables. Pearson chi-square tests were computed from raw, unweighted cell counts rather than rounded percentages, and Cramer's V was reported as an effect-size measure. Two-sided 95% Wilson score confidence intervals (Wilson, 1927) are reported for the proportions in Tables 2–4. Because the sample was purposively selected, these intervals should not be interpreted as population margins of error for Arab publics. Sensitivity analyses repeated the original chi-square tests after excluding the 130 comments whose gender presentation was undetermined.

**Table 2:** Emotional orientation by inferred public gender presentation.

Inferred presentation	Sympathetic	Unsympathetic	Unclear	Total
Male	370 40.5% [37.4, 43.7]	453 49.6% [46.4, 52.9]	90 9.9% [8.1, 12.0]	913 100%
Female	653 60.0% [57.0, 62.8]	319 29.3% [26.7, 32.1]	117 10.7% [9.0, 12.7]	1,089 100%
Undetermined	74 56.9% [48.3, 65.1]	39 30.0% [22.8, 38.4]	17 13.1% [8.3, 19.9]	130 100%
Total	1,097 51.5% [49.3, 53.6]	811 38.0% [36.0, 40.1]	224 10.5% [9.3, 11.9]	2,132 100%

Cells report *n*, row percentage, and pointwise 95% Wilson confidence interval. Pearson chi-square was calculated from raw counts: chi-square (4, *N* = 2,132) = 94.79, *p* < 0.001, Cramer's *V* = 0.15.

**Table 3:** Inferred commenter gender presentation within each case.

Coded case	Male	Female	Undetermined	Total
Case 1: Stigma/marginalization	549 50.8% [47.8, 53.8]	464 42.9% [40.0, 45.9]	68 6.3% [5.0, 7.9]	1,081 100%
Case 2: discrimination/injustice	138 18.1% [15.6, 21.0]	563 74.0% [70.7, 77.0]	60 7.9% [6.2, 10.0]	761 100%
Case 3: work/economic pressure	226 77.9% [72.8, 82.3]	62 21.4% [17.1, 26.5]	2 0.7% [0.2, 2.5]	290 100%
Total	913 42.8% [40.7, 44.9]	1,089 51.1% [49.0, 53.2]	130 6.1% [5.2, 7.2]	2,132 100%

Cells report *n*, row percentage, and pointwise 95% Wilson confidence interval. Pearson chi-square was calculated from raw counts: chi-square (4, *N* = 2,132) = 368.06, *p* < 0.001, Cramer's *V* = 0.29. The case-confounding qualifications in Table 1 apply.

**Table 4:** Dominant interpretive frame by inferred public gender presentation.

Inferred presentation	Religious	State/Institutional	Social	Victim-blaming	Unclear	Total
Male	429 47.0% [43.8, 50.2]	171 18.7% [16.3, 21.4]	136 14.9% [12.7, 17.4]	128 14.0% [11.9, 16.4]	49 5.4% [4.1, 7.0]	913 100%
Female	545 50.0% [47.1, 53.0]	220 20.2% [17.9, 22.7]	162 14.9% [12.9, 17.1]	101 9.3% [7.7, 11.1]	61 5.6% [4.4, 7.1]	1,089 100%
Undetermined	62 47.7% [39.3, 56.2]	29 22.3% [16.0, 30.2]	20 15.4% [10.2, 22.6]	8 6.2% [3.2, 11.7]	11 8.5% [4.8, 14.5]	130 100%
Total	1,036 48.6% [46.5, 50.7]	420 19.7% [18.1, 21.4]	318 14.9% [13.5, 16.5]	237 11.1% [9.9, 12.5]	121 5.7% [4.8, 6.7]	2,132 100%

Cells report *n*, row percentage, and pointwise 95% Wilson confidence interval. Pearson chi-square was calculated from raw counts: chi-square (8, *N* = 2,132) = 17.09, *p* = 0.029, Cramer's *V* = 0.06.

### Ethical considerations

The study analyzed only publicly accessible comments posted on public news pages. No private profiles, closed groups, direct messages, or restricted interactions were accessed. Usernames, profile images, hyperlinks, and potentially identifying details were removed from the working dataset. Results are reported in aggregate form, and no verbatim user-generated comments are reproduced in the manuscript, reducing traceability in a sensitive suicide-related context. The study did not contact users, infer individual mental health status, or attempt to identify commenters beyond the minimal public cues needed for aggregate coding.

Under applicable local legislation and institutional requirements, ethical review and approval were not required because the study analyzed only publicly accessible social media comments posted on public news pages, involved no interaction with users, and did not access private or restricted data.

## Results

### Sample overview

The final sample comprised 2,132 comments. Accounts inferred as female contributed 1,089 comments (51.1%), accounts inferred as male contributed 913 comments (42.8%), and 130 comments (6.1%) were classified as undetermined gender presentation. The undetermined category was retained to avoid imposing a binary classification where public naming cues were ambiguous.

### Inferred gender presentation and emotional orientation

Table 2 shows a statistically significant association between inferred commenter gender presentation and emotional orientation, chi-square(4, *N* = 2,132) = 94.79, *p* < 0.001, Cramer's *V* = 0.15. Comments from accounts inferred as female were coded sympathetic in 60.0% of cases (95% CI [57.0, 62.8]), compared with 40.5% among accounts inferred as male (95% CI [37.4, 43.7]). Comments from accounts inferred as male were more often coded unsympathetic (49.6%, 95% CI [46.4, 52.9]) than comments from accounts inferred as female (29.3%, 95% CI [26.7, 32.1]). The association is statistically clear but modest in magnitude, supporting H1 without implying a population-generalizable gender effect.

The pattern is more consistent with socially organized differences in emotional allocation than with a claim that one gender is inherently more compassionate. The observed distributions indicate that sympathy and condemnation were unequally expressed across publicly visible gender presentations within this corpus.

### Case-specific commenter composition

Table 3 shows that inferred commenter gender composition differed significantly across the three cases, chi-square(4, *N* = 2,132) = 368.06, *p* < 0.001, Cramer's *V* = 0.29, supporting H2 within this sample. In the Moroccan minor case, accounts inferred as female contributed 74.0% of comments (95% CI [70.7, 77.0]); in the employee case, accounts inferred as male contributed 77.9% (95% CI [72.8, 82.3]); and in the activist case, accounts inferred as male contributed 50.8% (95% CI [47.8, 53.8]) and accounts inferred as female 42.9% (95% CI [40.0, 45.9]). These differences describe the three case configurations and cannot isolate the effects of case topic, deceased gender, nationality, or media framing.

These differences indicate case-specific patterns of participation, but they should not be read as isolated effects of “context.” Each case combines a particular reported precipitating context with the deceased person's gender, nationality, public identity, and media framing. The result therefore identifies patterned participation across the three public cases rather than a causal estimate of the effect of sexual violence, stigma, or work pressure on commenter composition.

### Interpretive frames

Table 4 presents the dominant interpretive frames. Religious framing was most common across the dataset (48.6%, 95% CI [46.5, 50.7]), followed by state/institutional responsibility (19.7%, 95% CI [18.1, 21.4]), social explanations (14.9%, 95% CI [13.5, 16.5]), victim-blaming (11.1%, 95% CI [9.9, 12.5]), and unclear framing (5.7%, 95% CI [4.8, 6.7]). The association between inferred gender presentation and interpretive frame was statistically significant but weak, chi-square(8, *N* = 2,132) = 17.09, *p* = 0.029, Cramer's *V* = 0.06.

The examples in Table 5 illustrate the ambivalence of religious framing: religious language could express mercy and forgiveness or condemnation and denial of mercy. State and institutional comments placed responsibility on courts, police, employers, government services, media institutions, or mental health systems. Social comments emphasized family, customs, stigma, community values, gender inequality, and social cohesion. Victim-blaming comments placed responsibility primarily on the person who died. Emotional orientation and interpretive frame remain analytically separate categories.

**Table 5:** Paraphrased examples illustrating emotional orientation and interpretive frame.

ID	Dominant frame	Orientation	Non-verbatim English summary
E1	Religious	Sympathetic	Asks God to forgive the deceased and grant her peace.
E2	Religious	Unsympathetic	Restricts prayers for mercy to believers and withholds that prayer from the deceased.
E3	State/institutional	Unclear	Calls for enforceable labour protections and rejects unfair working conditions.
E4	Social	Sympathetic	Condemns the contempt and blame directed at a survivor of sexual violence.
E5	Victim-blaming	Unsympathetic	Questions the decision to die and says the employee should instead have left the job.

The examples are non-verbatim English summaries of distinct Arabic comments visible in the research archive. Names, photographs, direct links, and distinctive identifying details are omitted. The examples illustrate category boundaries and are not added to the quantitative counts.

### Sensitivity analyses

Excluding the 130 comments with undetermined gender presentation did not change the substantive direction of the findings. The association between inferred male/female presentation and emotional orientation remained significant, chi-square(2, *N* = 2,002) = 90.29, *p* < 0.001, Cramer's *V* = 0.21. Case-specific gender composition also remained strongly patterned, chi-square(2, *N* = 2,002) = 345.39, *p* < 0.001, Cramer's *V* = 0.42. The association between inferred male/female presentation and interpretive frame remained statistically significant but weak, chi-square(4, *N* = 2,002) = 11.33, *p* = 0.023, Cramer's *V* = 0.08. These sensitivity checks indicate that the small undetermined category does not account for the principal associations.

## Discussion

### Sympathy as a socially organized emotion

The central contribution of this study is to conceptualize public responses to suicide as a moral economy of sympathy. Commenters did not simply express private emotions after death; they publicly distributed compassion according to social rules about legitimate suffering, respectability, gendered vulnerability, religious worth, and institutional responsibility. This extends a sociology-of-emotions perspective by showing how sympathy can operate as a relational judgment whose object and intensity are socially regulated. In Hochschild's terms, the comment sections reveal implicit feeling rules concerning when mercy is appropriate, when indignation is redirected toward institutions, and when condemnation is treated as emotionally legitimate (Hochschild, 1979). Fassin's concept of moral economy further clarifies that these emotional judgments circulate within broader systems of values and obligations (Fassin, 2009).

### Gendered emotional allocation and case-specific participation

The first empirical finding concerns the association between inferred gender presentation and emotional orientation. Comments from accounts inferred as female were more frequently sympathetic, whereas comments from accounts inferred as male were more frequently unsympathetic. The magnitude of the omnibus association was modest (Cramer's *V* = 0.15), so the result should not be translated into a broad claim that women are inherently more compassionate than men. Instead, it is consistent with scholarship showing that gendered social positions can organize emotional involvement, help-giving, and attitudes toward suicidal behavior (Dahlen and Canetto, 2002; Eskin et al., 2016; Bezerra et al., 2022; Chen et al., 2025).

Commenter composition also varied substantially across cases (Cramer's *V* = 0.29), but those differences cannot be assigned to a single contextual factor. The Moroccan minor case drew a high proportion of comments from accounts inferred as female, whereas the workplace/economic-pressure case drew a high proportion from accounts inferred as male. Because each case also differs in the deceased person's gender, nationality, public identity, and media framing, these patterns are descriptive of the selected cases rather than evidence of an independent contextual effect.

### Religious ambivalence as an emotional repertoire

Nearly half of all comments used a religious interpretive frame, making religion the most visible moral vocabulary in the corpus. Yet religious framing did not operate in a single emotional direction. Because frame and emotional orientation were coded separately, the results show that the same broad religious repertoire could authorize mercy and prayer or condemnation and exclusion. This matters for the sociology of emotions because it demonstrates that feeling rules are not reducible to doctrine. Religious language supplies culturally available terms through which commenters can justify opposed emotional positions toward the deceased.

This ambivalence also advances the article's account of digital religion. In public comment cultures, religious meanings are not simply transferred from doctrine into online speech; they are enacted through platformed visibility, audience participation, and interactional norms (AlMazaedh et al., 2026). The comment section becomes an arena in which mercy, punishment, forgiveness, patience, and communal responsibility are publicly negotiated. Religious discourse can thus soften suicide stigma in some comments while intensifying postmortem exclusion in others.

### Digital death discourse and postmortem moral regulation

The second conceptual contribution concerns digital death discourse. These comment sections are not conventional spaces of mourning because many contributors may be strangers to the deceased, although relationships were not measured here. Klastrup (2015, 2018) and Harju (2015) show how dispersed publics participate in mediated mourning and remembrance, while Stokes (2015) emphasizes the ethical significance of digital remains. The present findings extend this discussion by showing that public death discourse can include not only sympathy and remembrance but also refusal of mercy, condemnation, and symbolic exclusion.

The activist case makes this form of postmortem moral regulation particularly visible. Some responses used sexuality, religion, national belonging, gender respectability, and perceived moral transgression as grounds for withholding compassion. In Bourdieu's terms, such judgments reproduce doxa by treating particular moral boundaries as self-evident and by determining whose suffering is recognized as legitimate (Bourdieu, 1991). The moral economy of sympathy is therefore also a mechanism of symbolic power: it distributes emotional recognition unevenly after death.

These judgments are also mediated by platform and news conditions. Facebook ranking and moderation shape which comments remain visible (Bucher, 2012; Gillespie, 2018), while platform selection more broadly organizes public visibility (van Dijck et al., 2018). The three cases were also presented through different media frames, summarized in Table 1, but framing is inseparable from case in this design. Because exact comment timestamps relative to each death were not retained, the study likewise cannot test whether emotional responses changed over time, a limitation that is especially relevant in light of Klastrup's (2018) emphasis on temporally differentiated mourning.

### Institutional blame and the social location of suffering

A substantial share of comments attributed responsibility to courts, government, employers, law enforcement, media, or mental health systems. This finding complicates any assumption that Arabic-language suicide discourse is uniformly religious or victim-blaming. Many commenters interpreted suicidal distress through failures of institutions, justice, social support, or economic regulation. In Durkheimian terms, such comments relocate suicide from individual pathology toward problems of social integration and regulation (Durkheim, 1951). In the moral economy developed here, sympathy can therefore be produced not only by identification with the deceased but also by transferring blame outward toward institutions perceived to have failed.

### Practical and theoretical implications

For journalists and social-media editors, the findings suggest that comment sections around suicide news can become sites of stigmatizing moral judgment, especially when the deceased was already socially marginalized. News organizations should avoid sensational framing, moderate dehumanizing or celebratory responses, and provide suicide-prevention and mental-health resources when reporting suicide. This recommendation is consistent with evidence on harmful and protective media-reporting effects (Niederkrotenthaler et al., 2010) and with suicide-related language guidance from the International Association for Suicide Prevention (n.d.). Mental-health advocates, educators, and religious or community leaders can also use the results to promote public language that recognizes suffering without romanticizing suicide and that resists denial of dignity to people who die by suicide or to their families.

For the sociology of emotions, the study demonstrates the value of digital comments as data on how emotional worth is organized in public life. Sympathy appears not as an unstructured reaction but as a socially patterned resource whose distribution is related to visible gender presentation, moral classification, religious vocabulary, and perceived institutional responsibility. The Arab digital setting is therefore not merely a culturally descriptive case. It provides a setting in which broader sociological questions about feeling rules, stigma, symbolic boundaries, public death, and the unequal distribution of compassion can be examined.

### Limitations and future research

Several limitations delimit the claims. First, the study analyzes three purposively selected cases and is not representative of Arab public opinion. Each case combines a particular individual, national setting, reported circumstance, and media framing, so cross-case differences cannot isolate the effect of any one attribute. Second, gender presentation was inferred from public naming cues; regional variation, ambiguous names, pseudonyms, shared accounts, and identities not captured by the coding scheme can produce misclassification.

Third, ranking, moderation, and deletion may affect which Facebook comments were visible at collection. Fourth, exact comment timestamps relative to the deaths were not retained, preventing temporal analysis. Finally, media framing is described at the case level but cannot be separated from the cases themselves, so the study does not estimate an independent framing effect.

## Conclusion

This study examined 2,132 Facebook comments on three suicide deaths discussed in Arabic-language digital spaces. Comments from accounts inferred as female were more often sympathetic than comments from accounts inferred as male in the pooled corpus, while religious discourse was the most common interpretive frame and operated as a language of both mercy and condemnation. Institutional and social frames also located suicide within questions of justice, work, stigma, law, and social support. These findings describe the selected cases and should not be generalized to Arab publics or interpreted as independent effects of gender, case context, or media framing.

The concept of a moral economy of sympathy moves the analysis beyond describing online reactions toward theorizing how digital publics regulate emotional recognition after suicide. Sympathy is not simply expressed; it is allocated through social feeling rules about legitimate suffering, moral worth, and responsibility. In Arabic-language Facebook comment cultures, suicide news becomes a public arena in which strangers negotiate who can be mourned, who can be blamed, and whose death can still command dignity.

## Data Availability

The raw data supporting the conclusions of this article will be made available by the authors, without undue reservation.
